# Development of cell combos micromethod to isolate respiratory viruses not detected by molecular techniques

**DOI:** 10.1128/spectrum.02571-25

**Published:** 2025-11-11

**Authors:** Margaux Valade, Marion Le Bideau, Clio Grimaldier, Céline Boschi, Philippe Colson, Bernard La Scola

**Affiliations:** 1 IHU Méditerranée Infection, Marseille, France; 2 Microbes Evolution Phylogeny and Infections (MEPHI), Aix-Marseille University 128791, Marseille, France; 3 Assistance Publique-Hôpitaux de Marseille (AP-HM), Marseille, France; Children's National Hospital, Washington, DC, USA

**Keywords:** respiratory virus, human infections, culture, virus isolation, cell combos

## Abstract

Virological diagnosis of respiratory infections relies mainly on molecular methods but can be overlooked in cases of unexpected or unknown emerging viruses. In this case, modern approaches use mainly metagenomics without *a priori*. The other approach to detecting new viruses is based on their isolation in cell culture using virus-specific cells and culture conditions. Unfortunately, inoculation on several lines and metagenomics requires a large volume of clinical sample, while the initial sample has already been largely used to carry out multiplexed RT-PCR. Herein, we aimed to develop micro-methods to inoculate combos of cell lines to isolate respiratory viruses missed by molecular techniques. Ten cell lines were selected, and then five cell combos of two cell lines each were developed. Strains of 15 respiratory virus species were inoculated, and viral growth was assessed by cytopathic effect detection and RT-PCR assays. Among these, all grew on at least one combo. The Caco-2/MRC5 combo was the most promising. As proof-of-concept, 859 human respiratory samples found negative by multiplex RT-PCR panels were inoculated into these five cell combos and monitored for the appearance of cytopathic effects. The efficiency of this approach was evidenced by isolating 12 herpes simplex or varicella-zoster viruses not detected by respiratory multiplex PCR assays. In conclusion, this updated approach can detect known but, most importantly, likely emerging respiratory viruses. It could be used to investigate undiagnosed respiratory infection outbreaks especially if optimized with an approach allowing for the detection of viral multiplications with minor or no cytopathic effects.

**IMPORTANCE:**

The detection of respiratory viruses relies on a range of laboratory methods each of which has distinct advantages in terms of speed, practicality, and sensitivity. Current molecular methods for respiratory virus detection, such as multiplex PCR, may fail to identify unexpected, genetically divergent, or emerging viruses. This study presents an innovative approach using micromethods inoculating combinations of cell lines (cell combos) to enhance the isolation of a broad panel of respiratory viruses, including those undetected by standard molecular techniques. This strategy revives and modernizes classical virology techniques for use in contemporary diagnostics, particularly during unexplained respiratory outbreaks. It opens up new possibilities for detecting both known and unknown viruses across different sample types.

## INTRODUCTION

Respiratory tract infections are among the most common diseases in humans and are an important cause of morbidity and mortality, particularly in vulnerable people (1). Most of these infections have viral etiologies (2). Thus, identifying the pathogens responsible for viral infection of the respiratory tract is important for reducing inter-human virus transmission, limiting the spread of epidemics, and preventing pandemics. It is also important for reducing hospitalizations and deaths, as well as proposing the most appropriate care and treatments. Viral detection can be fairly easy when the virus is already known because many assays are currently available that mainly consist of combinations of real-time PCR in multiplexed panels (3, 4). However, viruses can be overlooked as not targeted in cases where they are not expected to be involved in the disease or are unknown, emerging viruses. In such cases, modern approaches are based on viral metagenomics or metatranscriptomics using next-generation sequencing, the performance of which can be improved by viral enrichment using capture hybridization probes (5). Despite their efficiency in a few cases, these techniques lack sensitivity, mostly because of the strongly unfavorable ratio between viral and cellular nucleic acids, or because viruses remain untargeted, are genetically divergent, or are unknown. In such settings, detecting viruses by bringing out a cytopathic effect in culture and amplifying them by a culture step before sequencing are interesting approaches to potentially improve virus detection and identification in clinical samples and, possibly, to uncover new viruses (6). One limitation of this approach is that each virus has its own cell tropism through the use of specific receptors, co-receptors, and cellular machinery. Consequently, viruses do not grow on any cell line, and the spectrum of permissive cells and culture conditions can be narrow. Therefore, particularly with the goal of detecting and isolating potentially unexpected, rare, or new viruses, it is necessary to inoculate samples on a large panel of cell lines (7, 8). Such attempts are further hampered by the low number of clinical samples usually available for inoculation, as other diagnostic approaches are used in first line in clinical microbiology and virology laboratories. One solution could consist of inoculating samples on cell mats that combine different cell lines, and on reduced-in-size cell culture systems consisting of 96-well culture plates (9). Different commercial mixes of cells are available, such as R-Mix (Diagnostic Hybrids, Inc., Athens, OH), which combines mink lung cells (Mv1Lu) and human adenocarcinoma cells (A549), or H&V-Mix mixed FreshCells (Diagnostic Hybrids, Inc.), which combines monolayers of African green monkey kidney cells (CV-1) and human lung fibroblasts (MRC-5) (1, 9). The first mix was designed to target respiratory viruses and has been reported to successfully grow influenza A and B viruses, respiratory syncytial virus, parainfluenza viruses 1, 2, and 3, and adenoviruses (10). The second mix was designed to target herpes viruses.

Here, we aimed to develop micro-methods to inoculate different cell combos to allow the inoculation of low amounts of clinical respiratory samples on cell lines with different spectra of viral permissiveness in order to grow the broadest panel of respiratory viruses. Cell combos were first developed and validated using respiratory samples that tested positive for common respiratory viruses by PCR. As proof-of-concept, samples collected from patients with respiratory symptoms that tested negative using respiratory multiplex PCR were thereafter inoculated on these cell combos to attempt to isolate missed, unexpected, or new respiratory viruses.

## MATERIALS AND METHODS

### Cell lines and their combinations

Ten different cell lines were chosen through bibliographic searches to cover a broad range of permissive lineages for the isolation of respiratory viruses (Table 1). These cell lines were then paired to create five cell combos. MNT1 cells (ATCC-CRL-3450) were combined with H292 cells (ATCC-1848) to create the first combo, which we named MNT1/H292. Mv1Lu cells (ATCC-CCL-64) were associated with A549 cells (ATCC-CCL-185) to create the second combo, which we named Mv1Lu/A549. Ve6TMPRSS2 (NIBSC 100978) was associated with MDCK cells (ATCC-CCL-34) to create a third combo, which we named Ve6TMPRSS2/MDCK. Caco-2 cells (ATCC-HTB-37) were associated with MRC5 cells (ATCC-CCL-171) to create a fourth combo, which we named Caco-2/MRC5. Finally, BHK21 cells (ATCC-CCL-10) were combined with L929 cells (ATCC-CCL-1) to create the last cell combo, which we named BhK21/L929. Before the creation of these cell combos, all cell lines were cultivated individually in cell culture flasks (Dutscher, Brumath, France; ref. 003289) in their specific growth medium (the composition of which is described in Table 1) at 37 C under 5% CO_2_.

**TABLE 1:** List and characteristics of cell lines and culture media and permissive viruses^*a*^

Cell lines	Origin of cells	Composition of culture medium	Viruses that are permissive
MNT1	Lymph node of human	DMEM culture medium supplemented with 20% of fetal calf serum and 1% of AIM V culture medium	Parainfluenza virus and metapneumovirus (11, 12)
H292	Lung of human	RPMI culture medium supplemented with 10% of fetal calf serum and 1% of L-glutamine	Adenovirus, parainfluenza virus, RSV, and some strains of influenza virus and rhinovirus (13–15)
Mv1Lu	Lung of vison	MEM culture medium supplemented with 10% of fetal calf serum, 1% of L-glutamine, and 0,425 g of NAHCO3 powder	RSV, different strains of SARS-CoV-2, and influenza virus (16–18)
A549	Lung of human	DMEM culture medium supplemented with 10% fetal calf serum and 1% L-glutamine	Metapneumovirus, RSV, adenovirus, SARS-CoV-2, and influenza A virus (19–23)
Ve6TMPRSS2	Kidney of monkey and modified to constitutively express TMPRSS2	DMEM culture medium supplemented with 10% of fetal calf serum, 1% of L-glutamine, and 2% of Geneticin	SARS-CoV2 and HCoV-OC43 (24, 25)
MDCK	Kidney of dog	MEM culture medium supplemented with 10% of fetal calf serum and 1% of L-glutamine	Influenza viruses, adenovirus, parainfluenza virus, and RSV (26–29)
Caco-2	Large intestine and colon of human	DMEM culture medium supplemented with 10% of fetal calf serum, 1% of L-glutamine, and 1% of non-essential amino acids	Adenovirus, influenza virus, parainfluenza virus, HCoV-229E, metapneumovirus, and SARS-CoV-2 (8, 30)
MRC5	Lung of human	MEM culture medium supplemented with 10% of fetal calf serum and 1% of L-glutamine	Metapneumovirus, HCoV-229E, HCoVOC43, parainfluenza 1 virus, and influenza virus (31–34)
BHK-21	Kidney of hamster	MEM culture medium supplemented with 4% of fetal calf serum and 1% of L-glutamine	Metapneumovirus and HCoV-OC43 (35, 36)
L929	Subcutaneous connective tissue of mouse	MEM culture medium supplemented with 4% of fetal calf serum and 1% of L-glutamine	Influenza viruses (37)

^*a*^ HCoV, human coronavirus; HPIV, human parainfluenza virus; HRSV, human respiratory syncytial virus; SARS-CoV-2, severe acute respiratory syndrome coronavirus 2.

### Respiratory virus isolates

Different respiratory virus strains previously isolated from clinical samples and stored at −80°C in our laboratory were used in this study. They consisted of the following viral species: influenza A virus, influenza B virus, human parainfluenza (HPIV)−1, HPIV-2, HPIV-3, HPIV-4, rhinovirus, human respiratory syncytial virus (HRSV)-A, HRSV-B, human endemic coronavirus (HCoV)-OC43, HCoV-229E, severe acute respiratory syndrome coronavirus 2 (SARS-CoV-2) Wuhan strain, SARS-CoV-2 Omicron BA.1 variant, adenovirus, and metapneumovirus. These virus isolates were obtained retrospectively from stored residues of respiratory samples that had been sent to our clinical microbiology laboratory for diagnosis in the setting of routine clinical care, in the absence of any supplementary sampling.

### Clinical samples

Residues of respiratory samples used here had been stored at −80°C post analysis. They had been sent to our clinical microbiology laboratory for diagnosis in the setting of routine clinical care, in the absence of any supplementary sampling. They consisted of 859 samples that had been collected between December 2023 and June 2024. They were tested using the BioFire Respiratory Panel 2 plus multiplex PCR assay (FilmArray, bioMérieux, Marcy l’Etoile, France) with a negative result, indicating that no nucleic acids of infectious agents targeted in this assay were detected.

### Cell combo preparation

For each cell line cultivated in culture flasks, the medium was removed and 2.5 mL of trypsin (Thermo Fisher Scientific, Waltham, MA, USA; ref. 15050065) was added before incubating for 10 min at 37°C under 5% CO_2_. All cells were then peeled off and resuspended in their flask with 25 mL of medium specific to each combo (the composition of which is described in Table 2). Concentrations of each individual cell line were determined before the association of the two cell lines to create cell combos. For combo MNT1/H292, the two cell lines were associated with a concentration of 1.5 × 10^5^ cells/mL for MNT1 cells and 5 × 10^5^ cells/mL for H292 cells. For combo Mv1Lu/A549, the two cell lines were associated with a concentration of 4 × 10^5^ cells/mL for Mv1Lu cells and 2 × 10^5^ cells/mL for A549 cells. For combo Ve6TMPRSS2/MDCK, the two cell lines were associated with a concentration of 4 × 10^5^ cells/mL for Ve6TMPRSS2 cells and 2 × 10^5^ cells/mL for MDCK cells. For combo Caco-2/MRC5, the two cell lines were associated with a concentration of 2 × 10^5^ cells/mL for the Caco-2 cells and 4 × 10^5^ cells/mL for MRC5 cells. For combo BHK-21/L929, the two cell lines were associated with a concentration of 3 × 10^5^ cells/mL for BhK21 cells and 2 × 10^5^ cells/mL for L929 cells. For each combo, 96-well flat-bottom microplates were prepared and incubated at 37°C under 5% CO_2_ for 48 h before inoculation.

**TABLE 2:** List and characteristics of cell combos

Cell combo	Cell concentration	Composition of medium of culture
Combo MNT1/H292	MNT1: 1.5 × 10^5^ cells/mL and H292: 5 × 10^5^ cells/mL	DMEM culture medium supplemented with 10% fetal calf serum and 1% L-glutamine
Combo Mv1Lu/A549	Mv1Lu: 4 × 10^5^ cells/ml and A549: 2 × 10^5^ cells/mL	MEM culture medium supplemented with 10% of fetal calf serum, 1% of L-glutamine, and 0.425 g of NAHCO3 powder
Combo Ve6TMPRSS2/MDCK	Ve6TMPRSS2: 1 × 10^5^ cells/mL and MDCK: 3 × 10^5^ cells/mL	MEM culture medium supplemented with 10% of fetal calf serum and 1% of L-glutamine
Combo Caco-2/MRC5	Caco-2: 2 × 10^5^ cells/mL and MRC5: 4 × 10^5^ cells/mL	MEM culture medium supplemented with 4% of fetal calf serum and 1% of L-glutamine
Combo BHK-21/L929	BHK-21: 3 × 10^5^ cells/mL and L929: 2 × 10^5^ cells/mL	MEM culture medium supplemented with 4% of fetal calf serum and 1% of L-glutamine

### Inoculation of respiratory virus strains and culture on cell combos

For all virus strains, a 1:10 dilution in the growth medium specific to each cell combo was prepared individually. For each cell combo, medium was removed from 96-well flat-bottom microplates prepared 48 h before infection, and four wells were inoculated with 50 µL of the previously diluted viral strains. The plates were next centrifugated for 1 h at 2,000*g* at 37°C. Then, 200 µL of specific growth medium was added to all wells. For each virus, one of the four wells was sacrificed, as 200 µL was collected and stored at −80°C, which was day 0 of our test. Thereafter, the microplates were incubated at 37°C under 5% CO_2_ for 7 days, with daily observations using an EVOS FL digital inverted fluorescence microscope (Invitrogen, Carlsbad, CA, USA) at 10× magnification to detect the presence of cytopathic effects (CPE). After seven days of culture, the three remaining wells containing each virus were collected. All experiments were carried out in triplicate for each virus on each cell combo.

### Inoculation of clinical samples and virus culture on cell combos

For all respiratory samples, two conditions of culture were used on each cell combo. The first condition was the inoculation with a cocktail of antibiotics composed with fungizone (1 µg/mL), imipenem (5 µg/mL), gentamicin (2 µg/mL), colistin (10 µg/mL), and vancomycin (5 µg/mL). Culture medium was removed and two wells of 96-well microplates for each cell combo were inoculated with 50 µL of respiratory sample. The microplates were then centrifugated at 2,000*g* and 37°C for 1 h, and 200 µL of specific combo cell medium complemented with antibiotics was added to each well. The second condition was the filtration of respiratory samples prior inoculation. A total of 300 µL of samples was filtered on a 0.22 µm pore-sized centrifugal filter (Merck Millipore, Darmstadt, Germany). Then, medium was removed from each cell combo microplate well, and two wells were inoculated with 50 µL of the filtrate, and microplates were centrifuged at 2,000 × *g* and 37°C for 1 h. Thereafter, microplates were incubated at 37°C under 5% CO_2_ and observed daily using an EVOS FL digital inverted fluorescence microscope (Invitrogen) at 10× magnification to detect the presence of CPE. After 7 days of culture, if no CPE was detected, two blind subcultures were performed by scraping off the cellular mat from the microplate wells and collecting 50 µL of suspensions, which was re-inoculated on new cell combo microplates from which culture medium was removed. Finally, 200 µL of specific growth medium was added in each well, and microplates were incubated at 37°C under 5% CO_2_ for 7 days between each subculture.

### Nucleic acid extraction and PCR testing

To detect the presence of viral RNA or DNA in culture supernatants, DNA/RNA extraction was performed for all samples using the KingFisher Flex System (Thermo Fisher Scientific; ref. 5400610) prior testing with the FTD respiratory pathogens 21 assay (Fast Track Diagnosis, Luxembourg), a multiplex PCR panel that targets two virus species not targeted by the BioFire respiratory multiplex PCR assay, including human bocavirus and human parechovirus, and can detect human enterovirus and human rhinovirus separately. For all experiments, the mean of the cycle threshold values (Ct) of the qPCR for the three culture microplate wells on day 7 was compared with that of the Ct at day 0, and viral growth was considered if we observed a Ct decrease >3.3, which is a proxy of a one log_10_ variation of the viral load (38). For the sample in which a CPE was observed but the FTD multiplex respiratory PCR assay was negative, several PCR targeting microbial 16S DNA, bovine polyomavirus and orthoreovirus, herpes simplex virus (HSV), varicella-zoster virus (VZV), cytomegalovirus (CMV), measles virus, mumps virus, and *Chlamydia psittaci* were performed. If no pathogen was detected by these molecular assays, electronic microscopy and metagenomic analyses were performed.

## RESULTS

### Cell combo description

**Fig 1:**
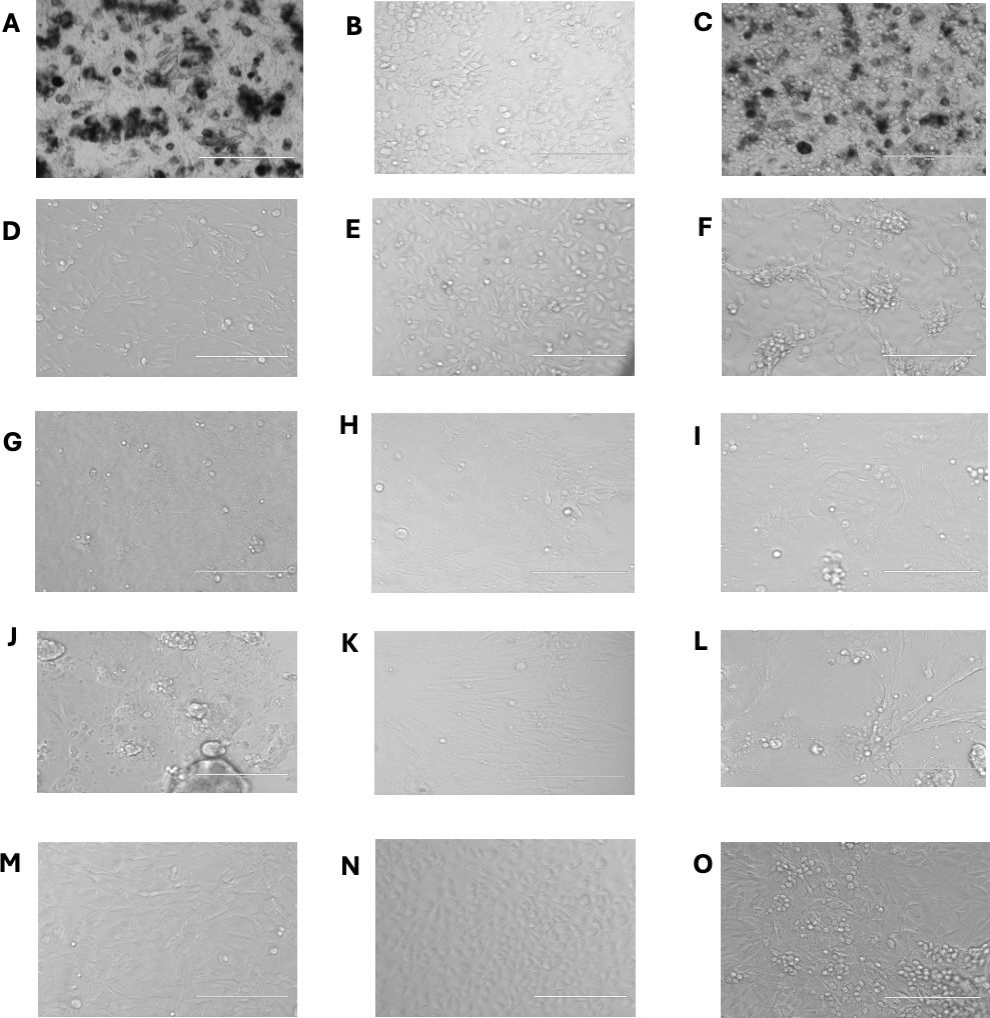
Images of cellular mats taken with an Evos FL digital inverted fluorescence microscope. (**A**) MNT1; (**B**) A549; (**C**) combo MNT1/H292; (**D**) Mv1Lu; (**E**) A549; (**F**) combo Mv1Lu/A549; (**G**) Ve6TMPRSS2; (**H**) MDCK; (**I**) ccombo Ve6TMPRSS2/MDCK; (**J**) Caco-2; (**K**) MRC5; (**L**) combo Caco-2/MRC5; (**M**) BHK-21; (**N**) L929; (**O**) combo BHK-21/L929. Pictures are taken at magnification ×20; scale bars indicate 400 µm.

Five cells combo were developed in our laboratory (Fig. 1). In cell combo MNT1/H292, the two cell lines were distinguishable in the same culture by the black color of MNT1, as these are highly pigmented human melanoma cells, whereas H292 cells are not pigmented. In combo Mv1Lu/A549, the two cell lines were distinguishable by their morphology, as Mv1Lu has an elongated morphology compared to A549 cells that have a rounded morphology. In combo Ve6TMPRSS2/MDCK, the two cell lines were distinguishable as MDCK cells form a network inside which Ve6TMPRSS2 cells exhibit a rounded morphology. In combo Caco-2/MRC5, the two cell lines were distinguishable by their morphology as MRC5 cells are fibroblasts with an elongated morphology, whereas Caco-2 cells are epithelial cells with a rounded morphology. Finally, in combo BHK21/L929, both cell lines were composed of fibroblasts, but BHK21 cells exhibit an elongated morphology, whereas L929 cells exhibit a rounded morphology.

### Inoculation with respiratory virus isolates

#### Cytopathic effect

**Fig 2:**
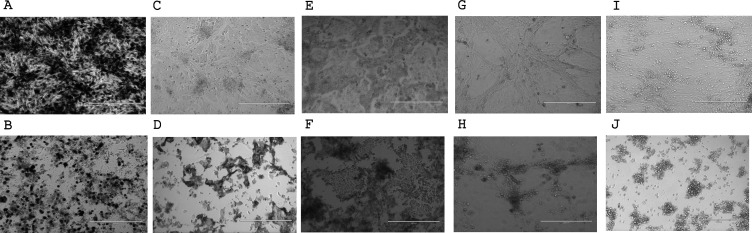
Examples of cytopathic effects with various viruses on each cell combo developed. (**A**) MNT1/H292 negative control, (**B**) HCoV-OC43 on MNT1/H292, (**C**) Mv1Lu/A549 negative control, (**D**) HRSV-B on Mv1Lu/A549, (**E**) Ve6TMPRSS2/MDCK negative control, (**F**) HPIV-1 on Ve6TMPRSS2/MDCK, (**G**) Caco-2/MRC5 negative control, (**H**) HCoV-229E on Caco-2/MRC5, (**I**) BHK-21/L929 negative control, (**J**) adenovirus on BHK-21/L929. Pictures are taken at magnification ×10; scale bars indicate 400 µm.

Daily observation of cell combos after virus inoculation allowed the detection of CPE for different viruses (Table 3; Fig. 2). On cell combos MNT1/H292 and Mv1Lu/A549, CPE was clear for HPIV-1, HPIV-2, HPIV-3, HRSV-A, HRSV-B, HCoV-OC43, and adenovirus. In contrast, no CPE was detected in these two cell combos for influenza B virus, rhinovirus, HCoV-229E, SARS-CoV-2 (Omicron variant), and metapneumovirus. For all other remaining viruses, CPE assessment was uncertain as it varied according to the observer. On cell combo Ve6TMPRSS2/MDCK, CPE was not detected with rhinovirus and HCoV-229E, was uncertain for HPIV-3 and metapneumovirus, and was detected for all other viruses. On cell combo Caco-2/MRC5, CPE was not detected for influenza B virus and rhinovirus, uncertain for influenza A virus, the four HPIV species, and metapneumovirus, and was detected for all other viruses. Finally, on combo BHK21/L929, CPE was detected for only two viruses (HPIV-2 and adenovirus), uncertain for HPIV-1, HPIV-3, HPIV-4, and HCoV-OC43, and was not detected for all other viruses.

**TABLE 3:** Cytopathic effects and viral growths on the five cell combos^*a*^

Viruses	Combo MNT1-H292	Combo Mv1Lu-A549	Combo VTMPRSS2-MDCK	Combo Caco-MRC5	Combo BHK21-L929
Influenza A virus	?/+	?/+	+/+	?/+	−/−
Influenza B virus	−/−	−/+	+/+	−/+	−/−
HPIV-1	+/+	+/+	+/+	?/+	?/+
HPIV-2	+/+	+/+	+/+	?/+	+/+
HPIV-3	+/+	+/+	?/+	?/+	?/+
HPIV-4	?/+	?/+	+/+	?/+	?/+
Rhinovirus	−/−	−/−	−/−	−/+	−/−
HRSV-A	+/+	+/+	+/+	+/+	−/−
HRSV-B	+/+	+/+	+/+	+/+	−
HCoV-OC43	+/+	+/+	+/+	+/+	?/+
HCoV-229E	−/+	−/+	−/−	+/+	−/−
SARS-CoV-2 (Wuhan strain)	?/+	?/+	+/+	+/+	−/−
SARS-CoV-2 (Omicron BA.1 strain)	−/−	−/−	+/+	+/+	−/−
Adenovirus	+/+	+/+	+/+	+/+	+/+
Metapneumovirus	−/−	−/−	?/+	?/+	−/−

^*a*^ HCoV, human coronavirus; HPIV, human parainfluenza virus; HRSV, human respiratory syncytial virus, SARS-CoV-2, severe acute respiratory syndrome coronavirus 2; +/+, presence of cytopathic effect and viral growth detected; ?/+, cytopathic effect uncertain and viral growth detected; −/+, absence of cytopathic effect and viral growth detected; −/−, absence of cytopathic effect and absence of viral growth.

#### RT-PCR for viral growth detection

**Fig 3:**
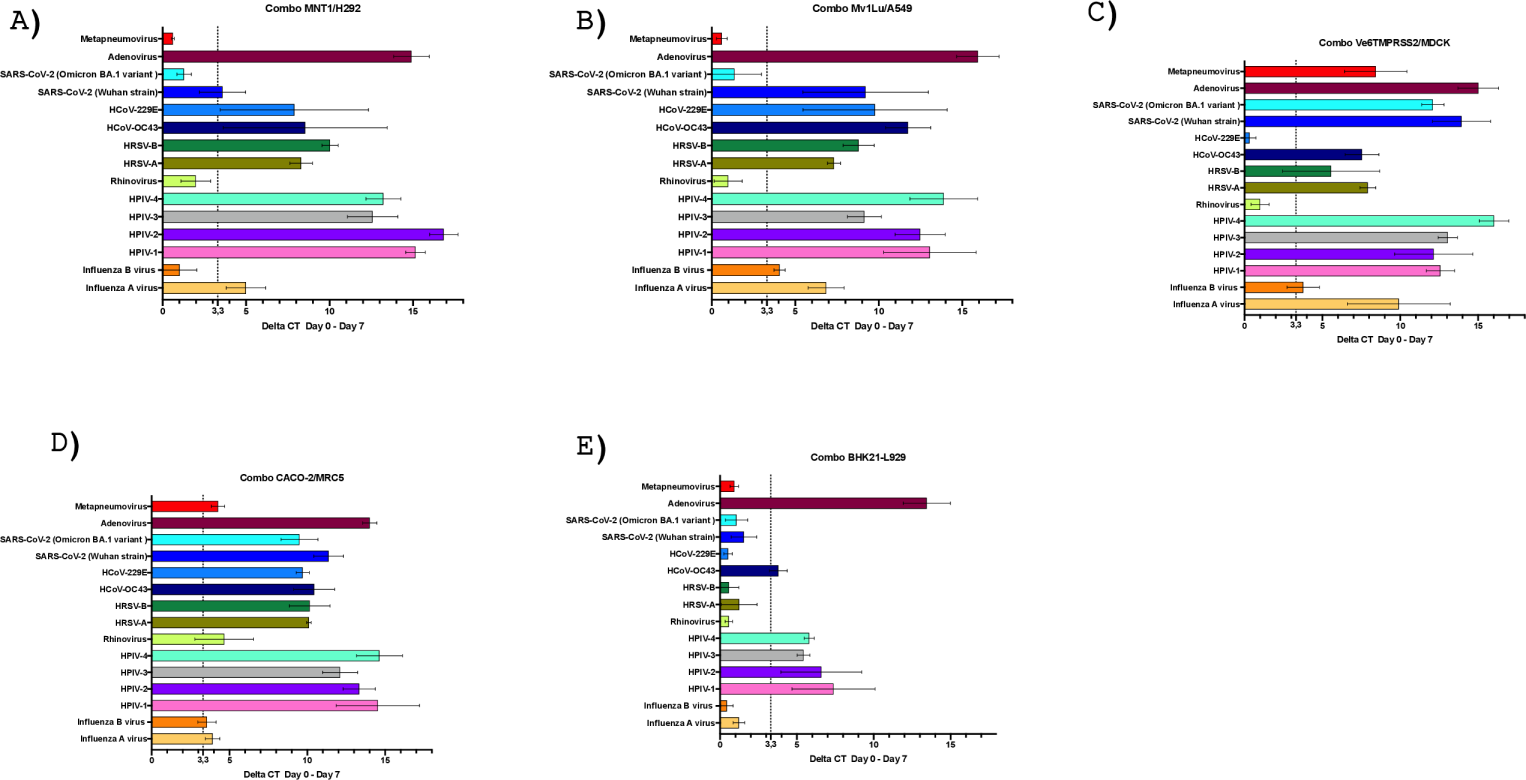
Respiratory virus growths on each cell combo developed. (**A**) Combo MNT1/H292; (**B**) combo Mv1Lu/A549; (**C**) combo VE6TMPRSS2/MDCK; (**D**) combo Caco-2/MRC5; (**E**) combo BHK21/L929. Dashed line indicates significant growth (3.3 Ct that is a proxy of a 1.0 log_10_ variation). Ct, real-time PCR cycle threshold value; HCoV, human coronavirus; HPIV, human parainfluenza virus; HRSV, human respiratory syncytial virus; SARS-CoV-2, severe acute respiratory syndrome coronavirus 2.

RT-PCR was performed on culture supernatants in parallel with CPE assessment. For combo MNT1/H292, PCR indicated viral growth (based on a >3.3 Ct decrease between days 0 and 7) for all but four viruses, namely influenza B virus, rhinovirus, SARS-CoV-2 (Omicron BA.1 variant), and metapneumovirus. For combo Mv1Lu/A549, PCR indicated viral growth for all but three viruses, namely rhinovirus, SARS-CoV-2 (Omicron BA.1 variant), and metapneumovirus. For combo Ve6TMPRSS2/MDCK, PCR indicated viral growth for all but two viruses, HCoV-229 and rhinovirus. For combo Caco-2/MRC5, PCR indicated the growth of all viruses. Finally, for combo BHK21/L929, PCR only indicated viral growth for the fourth HPIV, for HCoV-OC43, and for adenovirus (Fig. 3).

#### Comparison of the five cell combos

In the present study, the inoculation of 15 respiratory virus strains on the five combos revealed that, for each inoculated viral strain, viral growth was detected in at least one of the cell combos. The most promising cell combo was the Caco-2/MRC5, as all 15 viruses grew on it, whereas the least convincing was the BHK21/L929 combo, as viral growth was only detected for four viruses (approximately one-quarter of those tested). While cell combo Caco-2/MRC5 was the most promising, it was also the one for which CPE reading was the most difficult, since it was uncertain and varied according to the observer for six viruses (almost half of those tested). Parainfluenza virus and adenovirus were the only viruses for which viral growth was detected on the five cell combos. Culture was more difficult for three other viral strains tested, including rhinovirus that only grew on combo Caco-2/MRC5, and metapneumovirus and SARS-CoV-2 Omicron BA.1 that only grew on combos Caco-2/MRC5 and Ve6TMPRSS2/MDCK. If we consider the results of CPE detection and PCR, CPE detection was followed by PCR detection in all cases. Furthermore, viral growth was detected by PCR even if there was no CPE for three viruses including HCoV-229 on combos MNT1/H292 and Mv1Lu/A549, influenza B virus on combos Mv1Lu/A549 and Caco-2/MRC5, and rhinovirus on Caco-2/MRC5 (Table 3).

### Inoculation with clinical samples

As proof-of-concept, a total of 859 respiratory samples that previously tested negative with a respiratory multiplex PCR panel (BioFire Respiratory Panel 2.1 assay) were inoculated on the five combo cells. All samples were inoculated on combos MNT1/H292, Mv1Lu/A549, Ve6TMPRSS2/MDCK, and Caco-2/MRC5, while only 619 samples were inoculated on combo BHK21/L929, as it was implemented at a later stage. A CPE was detected on all cell combos for 12 respiratory samples. For these samples, culture supernatant was collected and tested by PCR using the FTD respiratory 21 multiplex PCR assay, which yielded negative results in all cases, and by simplex PCR assays, which retrieved positive results for HSV in 11 samples and for VZV in one sample.

After inoculation of the cell combos, supernatants were systematically collected to be tested by another respiratory multiplex PCR panel (FTD respiratory pathogens 21 assay) than the one used in the diagnosis setting. A total of 113 (13%) samples tested positive for various viruses, including for ≥2 viruses in 14 cases (Tables 4 and 5). Human bocavirus was detected in 12 cases, including co-infection in two cases, human parechovirus was detected in one case, and human enterovirus was detected in two cases, both as coinfections. These viruses are not targeted by the BioFire multiplex PCR assay and could not, therefore, be detected. In the 98 other samples for which a virus was detected, 11 different viruses that were not directly detected on the respiratory samples by the BioFire assay (although targeted) were detected in culture supernatants by the FTD multiplex PCR assay, including adenoviruses in 35 cases (co-infection in seven cases), human rhinovirus in 23 cases (co-infection in five cases), influenza A virus in 14 cases (co-infection in four cases), HCoV-HKU1 in 11 cases (co-infection in two cases), HCoV-229E in seven cases (co-infection in one case), HCoV-OC43 in six cases (co-infection in three cases), human metapneumovirus in four cases, RSV and HPIV-3 in four cases each, influenza B virus in two cases (in co-infection in both cases), HCoV-NL63 in two cases (in co-infection in one case), and HPIV-4 in one case. Excluding the detections of three viruses not targeted by the BioFire multiplex PCR assay, Ct were >30 for 86 (88%) of the 98 PCR detections, but were <25 in six cases, indicating higher viral loads. For these six latter samples, re-testing the nasopharyngeal sample by the BioFire assay made it possible to detect the virus identified by the FTD assay on the culture supernatant previously not detected.

**TABLE 4:** Respiratory viruses detected by qPCR in residues of respiratory clinical samples overall and cycle threshold values of qPCR^*a*^

Viruses detected	Ct	Number of samples
HBoV	19.8	1
HBoV	20.0	1
HBoV	23.3	1
HBoV	23.5	1
HBoV	25.7	1
HBoV	30.2	1
HBoV	31.6	1
HBoV	34.5	1
HBoV	>35.0	2
Human parechovirus	24.9	1
HPIV-3	24.6	1
HPIV-3	32.6	1
HPIV-3	33.8	1
HPIV-3	>35.0	1
HPIV-4	>35.0	1
Human adenovirus	33.5	1
Human adenovirus	33.6	1
Human adenovirus	34.4	1
Human adenovirus	34.8	1
Human adenovirus	>35.0	24
HRSV	22.0	1
HRSV	30.1	1
HRSV	31.0	1
HRSV	>35.0	1
Influenza A virus	25.4	1
Influenza A virus	31.0	1
Influenza A virus	34.4	1
Influenza A virus	>35.0	7
Human rhinovirus	26.9	1
Human rhinovirus	27.8	1
Human rhinovirus	27.8	1
Human rhinovirus	30.0	1
Human rhinovirus	31.1	1
Human rhinovirus	32.7	1
Human rhinovirus	32.8	1
Human rhinovirus	32.8	1
Human rhinovirus	34.5	1
Human rhinovirus	>35.0	9
Human metapneumovirus	18.2	1
Human metapneumovirus	33.4	1
Human metapneumovirus	33.5	1
Human metapneumovirus	>35.0	1
HCoV-229E	>35.0	6
HCoV-OC43	21.9	1
HCoV-OC43	30.0	1
HCoV-OC43	34.1	1
HCoV-NL63	29.5	1
HCoV-HKU1	>35.0	9

^*a*^ Ct, cycle threshold value of qPCR; HCoV, Human coronavirus; HPIV, human parainfluenza virus; HRSV, human respiratory syncytial virus, HBoV, human Bocavirus.

**TABLE 5:** Respiratory viruses detected by qPCR in coinfections

First virus	Ct	Second virus	Ct	Third virus	Ct
Human rhinovirus	27.1	HBoV	24.6		
Influenza B virus	34.4	Human rhinovirus	34.3		
HCoV-OC43	33.7	Human adenovirus	>35.0		
Influenza A virus	>35.0	Influenza B virus	>35.0		
Influenza A virus	>35.0	HBoV	>35.0	Human adenovirus	>35.0
HCoV-NL63	21.1	Human adenovirus	31.5		
Influenza A virus	19.9	HCoV-OC43	32.9		
Human rhinovirus	>35.0	HCoV-HKU1	30.4	Human enterovirus	31.2
Human rhinovirus	31.7	Human adenovirus	33.1		
HCoV-229E	>35.0	HCoV-OC43	>35.0		
Human enterovirus	34.0	Human adenovirus	34.3		
HCoV-HKU1	>35.0	Varicella-zoster virus	>35.0		
Human rhinovirus	>35.0	Human adenovirus	>35.0		
Influenza A virus	30.6	Human adenovirus	>35.0		

## DISCUSSION

Here, five cell combos were developed that made it possible to optimize the culture isolation of respiratory viruses by increasing both the throughput and the virus isolation rate while using a lower amount of clinical sample. This was assessed using large panels of viruses and clinical specimens, as they consisted in isolates of 15 different respiratory viruses and in 859 nasopharyngeal samples that tested negative by syndromic multiplex PCR assays. To the best of our knowledge, four of the five cell combos used here were not previously described while one has been previously reported and was commercialized.

Three previous studies compared the effectiveness of respiratory virus isolation from clinical samples using this commercial Mv1Lu/A549 kit with that of cultures with single cell types. The first study reported in 2000 a comparison of the efficacy of culture isolation of seven respiratory viruses (influenza A and B, adenovirus, RSV, HPIV1, HPIV2, and HPIV3) from nasopharyngeal aspirates using either the commercial mix culture Mv1Lu/A549 or a single cell type including Mv1Lu, A549, as well as Hep-2 and pRhMK. All viruses detected in single cell culture were also detected by an indirect immunofluorescent assay (IFA) in mixed culture Mv1Lu/A549, except for the case of one RSV-positive sample that was only positive on Hep-2 cells. These data demonstrated a similar capacity to detect multiple respiratory viruses for the cell combo Mv1Lu/A549 and cell lines used separately (39). In 2001, Barenfanger et al. confirmed these results by comparing combo Mv1Lu/A549 with Hep-2, MRC5, and pRhMK, used separately. Moreover, the time to positive culture as detected by CPE or hemadsorption-IFA was shorter with Mv1Lu/A549 (1.4 days) than with conventional, single cell line-based culture (5.2 days) (10). A third report dating from 2008 confirmed these two previous studies as the Mv1Lu/A549 combo was deemed more sensitive and rapid than single cell-based culture with MDCK, LLC-MK2, or HEp-2. All these data indicated that the commercial combo Mv1Lu/A549 was equally or even more sensitive than inoculation on a single cell line and allowed a faster detection, by several days. In the present study, cell combo Caco-2/MRC5 was the most promising, as all viral isolates grew on it. Several viruses, including rhinovirus, metapneumovirus, and SARS-CoV-2 Omicron BA.1, are difficult to cultivate and did not grow on most of the cell combos tested. The difference observed here between the viral growth of the two SARS-CoV-2 variants had already been reported at our laboratory as three Omicron strains did not grow using Mv1LU cells (16).

CPE assessment to detect viruses in clinical samples is an issue when using cell combos. Such assessment is subjective and may depend on the observer. Moreover, some viruses, including influenza virus, rhinovirus, metapneumovirus, SARS-CoV-2, and RSV, do not induce CPE. Also, HCoV-229E was reported to induce poorly legible or no CPE (40). Here, for three viruses, including HCoV-229E, influenza B virus, and rhinovirus, no CPE was observed although viral growth was detected by qPCR. Thus, even in the absence of CPE, various techniques including PCR or immunofluorescence can make it possible to detect viral growth (41–43), which warrants using them on culture supernatants even in the absence of CPE. As a matter of fact, the possibility of viral growth in the absence of CPE is one limitation of the present approach, as it can lead to overlooking the presence of viruses that are unknown and will, therefore, not be detectable using specific, targeted techniques such as PCR or immunofluorescence. This potential bias warrants the identification of still more “open” methods to detect viral presence.

In the present study, respiratory viruses not detected in 113 respiratory samples by the BioFire multiplex PCR assay were retrospectively detected by the FTD multiplex PCR assay. This may be explained by the fact that the FTD system targets two viruses, namely bocavirus and parechovirus that are not targeted by the Biofire assay, and distinguishes Rhinovirus and Enterovirus, whereas the BioFire assay does not. Alternatively, the FTD assay may be more sensitive than the BioFire assay in some cases, as 88% of the samples that tested positive but were previously missed by the BioFire assay exhibited a Ct >30. Besides, two other viruses, namely herpes simplex virus (HSV) and varicella-zoster virus, which belong to the same family *Herpesviridae* (44), were detected using different, specific PCR assays following detection of a CPE. Both viruses cause cutaneous lesions including orofacial ones and may be present in the respiratory tract. HSV is also known to infect a large panel of cell lines (45).

Overall, cell combos such as those implemented here are promising tools to detect known and possibly unknown respiratory viruses and could be used at least in particular settings, such as for the investigation of clusters or outbreaks of undiagnosed respiratory infections. Future studies incorporating comparisons with metagenomics could help establish the unique diagnostic contribution of the cell combo approach, particularly in identifying rare, unexpected, or novel viruses that may be missed by PCR panels. However, ideally, cell combos and next-generation sequencing-based approaches would be used in combination since they should be complementary tools as broadly observed for the case of microorganisms when comparing culturomics versus metagenomics (46). Furthermore, such combo cell-based approaches could be improved over time by using new cell lines and new combinations of cell lines in order to enhance the detection of the same or other respiratory viruses. Recently, a study reported that MA104 cells, which are epithelial cells from the kidney of an African green monkey, were permissive to bocavirus that has not been isolated to date using other cells (47), and it would be interesting to include this cell line in existing or new cell combos.

## Data Availability

Unreported data may be provided from the corresponding author upon reasonable requests.
